# Long-Travel 3-PRR Parallel Platform Based on Biomimetic Variable-Diameter Helical Flexible Hinges

**DOI:** 10.3390/mi15030338

**Published:** 2024-02-28

**Authors:** Hao Dong, Pengbo Liu, Shuaishuai Lu, Peng Yan, Qiyuan Sun

**Affiliations:** 1 School of Mechanical Engineering, Qilu University of Technology (Shandong Academy of Sciences), Jinan 250353, China; donghao19970424@163.com (H.D.); luss_me@qlu.edu.cn (S.L.); sunqiyuan0907@163.com (Q.S.); 2 Shandong Institute of Mechanical Design and Research, Jinan 250031, China; 3 Key Laboratory of High-Efficiency and Clean Mechanical Manufacture, Ministry of Education, School of Mechanical Engineering, Shandong University, Jinan 250061, China; yanpeng@sdu.edu.cn

**Keywords:** 3-PRR, 3-DOF planar parallel platform, flexible hinge, large workspace, kinematic analysis

## Abstract

Technological advancements across various sectors are driving a growing demand for large-scale three-degree-of-freedom micro–nano positioning platforms, with substantial pressure to reduce footprints while enhancing motion range and accuracy. This study proposes a three-prismatic-revolute-revolute (3-PRR) parallel mechanism based on biomimetic variable-diameter helical flexible hinges. The resulting platform achieves high-precision planar motion along the X- and Y-axes, a centimeter-level translation range, and a rotational range of 35° around the Z-axis by integrating six variable-diameter flexible helical hinges that serve as rotational joints when actuated by three miniature linear servo drives. The drives are directly connected to the moving platform, thereby enhancing the compactness of the system. A kinematic model of the motion platform was established, and the accuracy and effectiveness of the forward and inverse kinematic solutions were validated using finite element analysis. Finally, a prototype of the 3-PRR parallel platform was fabricated, and its kinematic performance was experimentally verified visually for improved endpoint displacement detection. The assessment results revealed a maximum displacement error of 9.5% and confirmed that, judging by its favorable workspace-to-footprint ratio, the final system is significantly more compact than those reported in the literature.

## 1. Introduction

Rapid advancements in precision optics, biotechnology, genetic engineering, microscopy, and aerospace technology have driven an increasing demand for innovative transmission devices capable of delivering ultra-high-precision motion over wide ranges. For instance, wafer alignment requires that the mechanism be adjusted to achieve ultra-high-precision pose adjustment with three degrees of freedom in the centimeter-level motion range. The linear and rotary accuracy requirements are at the sub-micron and micro-arc levels, respectively [1,2,3]. Similarly, in biomedical research, multi-degree-of-freedom precision manipulation platforms are required to achieve intricate operations at the cellular and even molecular levels within a centimeter-scale motion range [4,5,6]. In confocal microscopy, precision motion platforms are essential for positioning samples with micron-level accuracy within a centimeter-scale range [7]. Multi-degree-of-freedom precision transmission devices are also required for extensive beam pointing control in deep space exploration technology [8,9]. Thus, the applications for ultra-high-precision wide-range actuators are nearly endless.

Traditional mechanical transmission systems use rigid motion pairs to transfer forces and displacements, inevitably leading to clearance (and thus slight tolerance), friction, and wear between components. By contrast, in flexible mechanisms, motion relies on the elastic deformation of components, achieving force transmission and energy conversion concurrently with deformation. Compared to rigid mechanisms, flexible mechanisms offer both structural and functional advantages, including (1) structural simplicity that results in high reliability and suitability for integral design and processing; (2) absence of motion pair clearance that enables high-precision movement; (3) avoidance of friction and wear that minimize noise; (4) minimal lubrication requirements, which avoids system contamination; (5) potential overall stiffness variation that enables impact and vibration reduction and enhanced system adaptability to the environment; and (6) ability to store and convert energy, which improves transmission efficiency, thereby addressing original motion limitations.

Micromanipulation mechanisms for certain applications, such as wafer positioning, micro–nano manipulation, nanoimprint lithography production, and optical alignment, have stringent requirements regarding degrees of freedom and overall size [10]. Owing to their superior precision, flexible mechanisms have the potential for fulfilling such demands. Three-degree-of-freedom flexible parallel mechanisms have experienced significant development in the past few decades, especially in achieving large-scale motion. To achieve planar three-degree-of-freedom motion, these mechanisms can implement one of three possible parallel kinematic configurations: three-prismatic-revolute-revolute (3-PRR), three-prismatic-prismatic-revolute (3-PPR), and three-revolute-revolute-revolute (3-RRR) [11,12].

Numerous studies have explored multi-degree-of-freedom flexible mechanisms, often focusing on decoupling axes, reducing size, and increasing payload. In [13], a three-degree-of-freedom 3-RRR-type planar macro–micro composite precision positioning platform was presented. For macro-motion, this design uses traditional servo motor-driven rigid motion pairs, while micro-motion is achieved through a compliant mechanism driven by piezoelectric ceramics. A disadvantage of this design is the large footprint required by the macro-motion component to achieve extensive motion. In [14], a 3-PPR parallel platform driven by voice coil motors was presented, enhancing motion accuracy by incorporating a flexible four-bar mechanism. Notably, the rotational joint reduces the shaft drift and increases the rotation range without increasing the size of the flexible mechanism. In [15], a design was presented for an XYƟ planar flexible mechanism that achieves long travel ranges and high accuracy, wherein four symmetric voice coil motors drive R- and π-shaped spring plates to accomplish three-degree-of-freedom motion. Although a translational range of ±2 mm and rotational range of ±1.2° were achieved, the mechanism is not compact, and the use of four motors to control three degrees of freedom results in a relatively complex control algorithm. In [16], a three-degree-of-freedom large-angle tilt mechanism was designed, aiming to achieve a larger workspace (the z-axis translation is 10.98 mm, and rotation around the x and y axes is 4.7°) In this design, a decoupling mechanism effectively reduces the lateral displacement of the voice coil motor, thereby preventing contact with the stator during rotation. In [17], an I-shaped flexible hinge was used to decouple the motion of a three-degree-of-freedom flexible mechanism in the XYZ direction, achieving approximately 2 mm of travel in each direction. In [18], a flexible hinge made of a shape-memory alloy with optimized notch shapes was presented, which provides ±5° motion within a circular workspace with a radius of 10 mm. In [19], a six-degree-of-freedom macro–micro compliant precision motion platform was designed. Although the mechanism is reportedly capable of achieving a maximum linear motion of 10 mm and a maximum rotational range of 6°, it faces various practical engineering challenges, such as uncertainties in motion conversion between macro- and micro-movements. Therefore, despite significant existing research, the design of a precision positioning platform with no clearances and a wide range of motion remains a challenge, especially in single motion mode.

From the literature, it is evident that achieving wider ranges of motion has thus far required mechanisms with significantly larger overall dimensions than their lower-range counterparts. In addition, the typically limited range of motion of flexible hinges is a key limiting factor in flexible mechanisms. Therefore, designing flexible hinges with a wide range of motion holds promise for enhancing the range of motion of flexible mechanisms, enabling large-scale and high-precision movements [20].

In this study, a three-degree-of-freedom planar flexible parallel mechanism was designed using a 3-PRR configuration. In this configuration, P refers to an active joint that serves as the motion input, whereas R stands for two passive rotational joints comprising two biomimetic variable-diameter helical flexible hinges. The 3-PRR configuration was chosen because, unlike the other two configurations (3-PPR and 3-RRR), it provides wide travel ranges with good decoupling and isotropy, thus enabling the mechanism to achieve high precision and a wide range of motion. The remainder of this paper is organized as follows: Section 2 introduces the mechanical design of the proposed 3-PRR flexible parallel motion platform, Section 3 establishes the forward and inverse kinematic models for the platform, and Section 4 presents the fabrication of a prototype platform along with simulation and experimental results that validate the theoretical kinematic model and the feasibility of using simulations to control the physical system.

## 2. Materials and Methods

### 2.1. Mechanism Design

Traditionally, platforms achieving multiple degrees of freedom within a plane rely on multiple drivers that actuate complex transmission and amplification mechanisms. However, this approach significantly increases the overall platform size, thereby limiting its operational range. Flexible hinges, on the other hand, have the potential to achieve large displacements through structural design or the use of highly specific deformable materials. Recognizing this potential, we propose a 3-PRR flexible parallel motion platform based on a biomimetic variable-diameter helical flexible hinge. This design aims to provide a compact solution to accomplish ultra-precise 3-DOF planar actuation with a substantial range of motion.

#### 2.1.1. Variable-Diameter Helical Flexible Hinge

**Figure 1:**
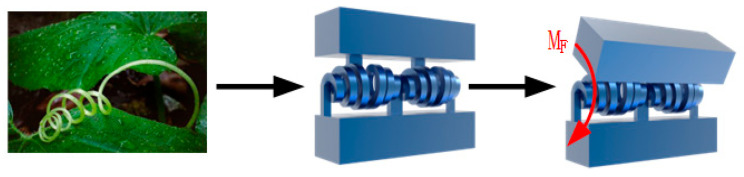
Principle of spiral motion.

**Figure 2:**
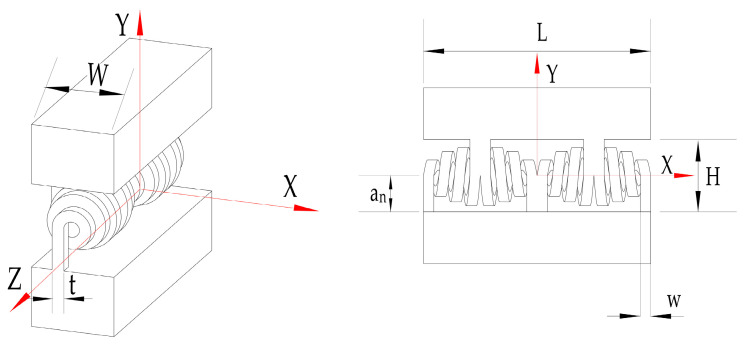
Schematic diagram of flexible hinge. The distance between the two rigid bodies is denoted by H, whereas the length and width of each rigid body are represented by L and W, respectively. The spiral unit has a square cross section of length w, width t, and twist arm length a_n_.

To achieve a wide rotation range, we propose the bio-inspired variable-diameter spiral flexible hinge illustrated in Figure 1. Drawing inspiration from naturally occurring spirals found in vines, our design incorporates the Archimedean spiral curve. This hinge is capable of extensive rotational motion (up to 90°) while limiting parasitic displacement and constraining drift along the axis of rotation. The hinge was designed for additive manufacturing and serves as the rotational component in the proposed motion mechanism, facilitating the continuous transmission of motion and force. As depicted in Figure 2, the flexible hinge comprises two biomimetic spiral units arranged in a series and linked to two rigid bodies by five flexible beams. Table 1 lists the key dimensions of the evaluated flexible hinge, with the naming conventions corresponding to those shown in Figure 2.

#### 2.1.2. Design of the 3-PRR Flexible Parallel Platform

**Figure 3:**
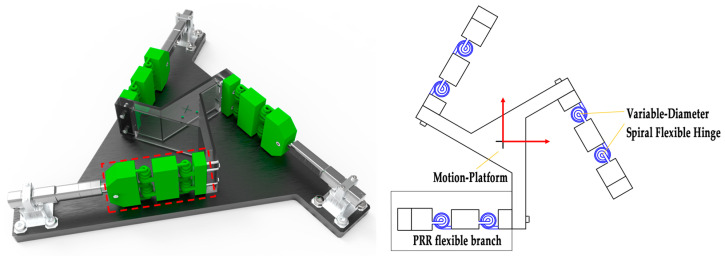
3-PRR flexible parallel platform.

A strategic arrangement of the proposed flexible spring structures can achieve a smaller footprint than that of traditional planar spring structures, resulting in higher spatial efficiency and reduced shaft drift while enabling extensive rotation. We constructed a 3-PRR flexible parallel platform composed of six of the proposed biomimetic variable-diameter spiral flexible hinges, as shown in Figure 3. The parallel platform consists of three motion branches and an end-effector motion platform. The three 3-PRR flexible branches are symmetrically distributed in rotation at intervals of 120° around the center of the moving platform. Each PRR flexible branch includes two flexible rotational joints (designed as R-joints using the proposed variable-diameter spiral-flexible hinges) and one flexible translational joint. Force and motion in each branch are initially input by a micro linear servo drive. Under the influence of the driven translational elements, the flexible hinges undergo elastic deformation, ultimately resulting in three-degree-of-freedom displacement of the platform. This configuration ensures more deterministic motion with minimal parasitic movement compared to traditional designs [14].

### 2.2. Kinematic Model

**Figure 4:**
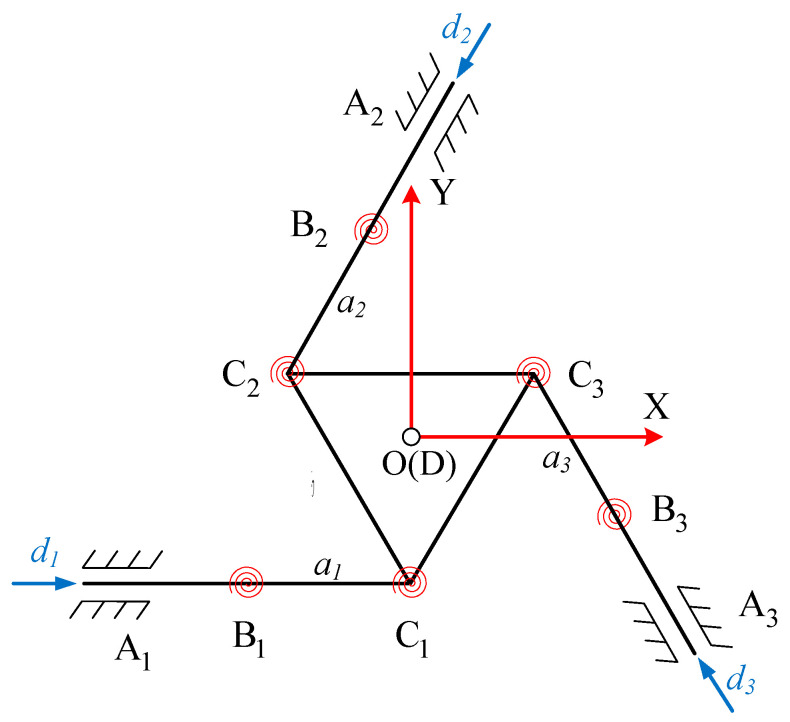
Pseudo-rigid body model of the planar 3-PRR flexible parallel mechanism.

Figure 4 shows the pseudo-rigid body model of the proposed 3-PRR flexible parallel mechanism. As described above, the proposed biomimetic variable-diameter spiral flexible hinges in the parallel mechanism serve as joints. Therefore, in kinematic modeling, these rotational components are treated as ideal hinges, simplifying the kinematics.

The platform is fixed at *A*_1_, *A*_2_, and *A*_3_, with rods *A*_1_*B*_1_, *A*_2_*B*_2_, and *A*_3_*B*_3_ serving as the corresponding translational joints input into the mechanism. The respective rotational joints are represented by *B_i_* (*i* = 1, 2, 3) and *C_i_* (*i* = 1, 2, 3), and the lengths of rods *B*_1_*C*_1_, *B*_2_*C*_2_, and *B*_3_*C*_3_ are denoted as *a*_1_, *a*_2_, and *a*_3_, respectively. The flexible hinges *C*_1_, *C*_2_, and *C*_3_ are connected to the moving platform, forming an internal triangular structure with side length *b*. For analytical convenience, the initial position of the geometric center point *D* of this triangle coincides with the defined coordinate origin *O*. The entire mechanism comprises three PRR flexible branches arranged at intervals of 120°. The input displacements of rods *A*_1_*B*_1_, *A*_2_*B*_2_, and *A*_3_*B*_3_ are represented by *d*_1_, *d*_2_, and *d*_3_, whereas the output displacements are the coordinates of point *D*. In this case, the platform’s angular displacements (X_D_, *Y_D_*, and *φ*) serve as the output displacements. The coordinates of points *B_i_* (*i =* 1, 2, 3) given by *X_Bi_* (*i* = 1, 2, 3) and *Y_Bi_* (*i =* 1, 2, 3), in their initial condition, can be expressed as follows:

(1) $$B_{1}\left(-a_{1},-\frac{\sqrt{3}}{3}b\right),B_{2}\left(\frac{a_{2}-b}{2},\frac{3\sqrt{3}a_{2}+\sqrt{3}b}{6}\right),B_{3}\left(\frac{a_{3}+b}{2},\frac{-3\sqrt{3}a_{3}+\sqrt{3}b}{6}\right),$$

#### 2.2.1. Inverse Kinematics Solution

**Figure 5:**
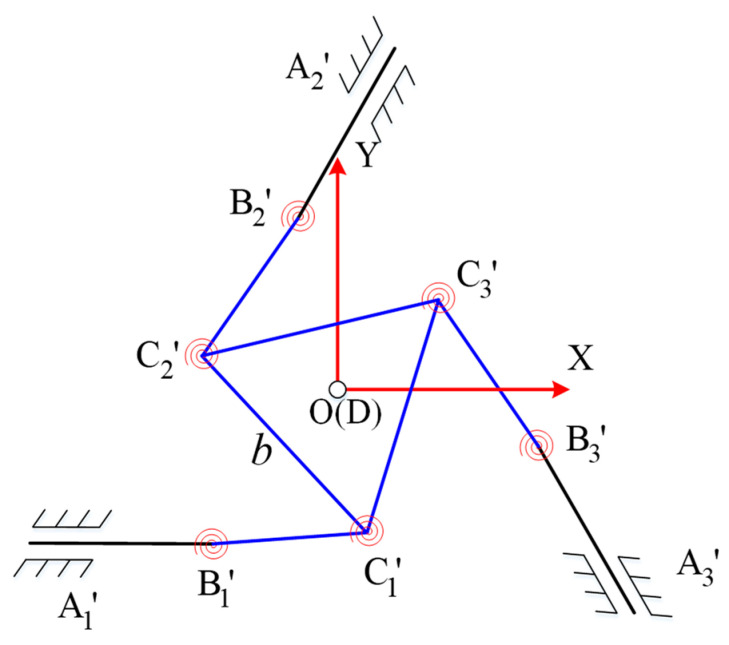
Kinematic computational model of the planar 3-PRR flexible parallel mechanism.

As shown in Figure 5, providing a certain input displacement (*d*_1_, *d*_2_, and *d*_3_) for the three branches causes deformation in each rotational joint, placing each joint at a new coordinate with respect to the moving platform. When a new target position is set as the geometric center of the moving platform, a series of equations are solved to determine the required displacements (*d*_1_, *d*_2_, and *d*_3_) along the three axes of the moving platform. This process is known as inverse kinematic analysis of the mechanism. The solving process begins by establishing the expressions for the coordinates of the relevant points after the planned movement has occurred, as expressed in Equations (2)–(7).

The coordinates of the new position *B*_1′_ of point *B*_1_ can be expressed as

(2) $$\begin{array}{l} X_{B_{1}^{\prime }}=-\left(a_{1}-d_{1}\right)\\ Y_{B_{1}^{\prime }}=-\frac{\sqrt{3}}{3}b \end{array}$$

Similarly, the new coordinates *C*_1′_, *B*_2′_, *C*_2′_, *B*_3′_, and *C*_3′_ of *C*_1_, *B*_2_, *C*_2_, *B*_3_, and *C*_3_, respectively, can be expressed as:

(3) $$\begin{array}{c} X_{C_{1}^{\prime }}=X_{D}+\frac{\sqrt{3}}{3}b\sin\phi \\ Y_{C_{1}^{\prime }}=Y_{D}-\frac{\sqrt{3}}{3}b\cos\phi \end{array}$$

(4) $$\begin{array}{c} X_{B_{2}^{\prime }}=X_{B_{2}}-\frac{d_{2}}{2}\\ Y_{B_{2}^{\prime }}=Y_{B_{2}}-\frac{\sqrt{3}}{2}d_{2} \end{array}$$

(5) $$\begin{array}{c} X_{C_{2}^{\prime }}=X_{C_{1}^{\prime }}-b\cos\left(60^{{}^{\circ}}-\phi \right)\\ Y_{C_{2}^{\prime }}=Y_{C_{1}^{\prime }}+b\sin\left(60^{{}^{\circ}}-\phi \right) \end{array}$$

(6) $$\begin{array}{c} X_{B_{3}^{\prime }}=X_{B_{3}}-\frac{d_{3}}{2}\\ Y_{B_{3}^{\prime }}=Y_{B_{3}}+\frac{\sqrt{3}}{2}d_{3} \end{array}$$

(7) $$\begin{array}{c} X_{C_{3}^{\prime }}=X_{C_{1}^{\prime }}+b\cos\left(60^{{}^{\circ}}+\phi \right)\\ Y_{C_{3}^{\prime }}=Y_{C_{1}^{\prime }}+b\sin\left(60^{{}^{\circ}}+\phi \right) \end{array}$$

Since rod *B_i_C_i_* (*i* = 1, 2, 3) is regarded as a rigid member (the deformation after loading is extremely small and can be neglected), the rod length constraints should be satisfied before and after motion. Hence,

(8) $${\left(X_{C_{i}^{\prime }}-X_{B_{1}^{\prime }}\right)}^{2}+{\left(Y_{C_{i}^{\prime }}-Y_{B_{1}^{\prime }}\right)}^{2}={a_{i}}^{2}$$

Collating Equations (2)–(8) yields

(9) $$\begin{array}{r} {\left(A_{1}-d_{1}\right)}^{2}+{B_{1}}^{2}={a_{1}}^{2}\\ {\left(A_{2}+\frac{d_{2}}{2}\right)}^{2}+{\left(B_{2}+\frac{\sqrt{3}d_{2}}{2}\right)}^{2}={a_{2}}^{2}\\ {\left(A_{3}+\frac{d_{3}}{2}\right)}^{2}+{\left(B_{3}-\frac{\sqrt{3}d_{3}}{2}\right)}^{2}={a_{3}}^{2} \end{array}$$

where:

(10) $$\begin{array}{l} A_{1}=X_{D}+\frac{\sqrt{3}}{3}b\sin\phi +a_{1}\\ B_{1}=Y_{D}-\frac{\sqrt{3}}{3}b\sin\phi +\frac{\sqrt{3}}{3}b\\ A_{2}=X_{D}+\frac{\sqrt{3}}{3}b\sin\phi -b\cos\left(60^{{}^{\circ}}-\phi \right)-\frac{a_{2}-b}{2}\\ B_{2}=Y_{D}-\frac{\sqrt{3}}{3}b\sin\phi +\frac{\sqrt{3}}{3}b+b\sin\left(60^{{}^{\circ}}-\phi \right)-\frac{3\sqrt{3}a_{2}+\sqrt{3}b}{6}\\ A_{3}=X_{D}+\frac{\sqrt{3}}{3}b\sin\phi +b\cos\left(60^{{}^{\circ}}+\phi \right)-\frac{a_{1}+b}{2}\\ B_{3}=Y_{D}-\frac{\sqrt{3}}{3}b\sin\phi +\frac{\sqrt{3}}{3}b+b\sin\left(60^{{}^{\circ}}+\phi \right)-\frac{\sqrt{3}b-3\sqrt{3}a_{2}}{6} \end{array}$$

Solving Equation (9) yields

(11) $$\begin{array}{l} d_{1}=A_{1}-\sqrt{a_{1}-B_{1}}\\ d_{2}=\frac{-\left(A_{2}+\sqrt{3}B_{2}\right)-\sqrt{-3{A_{2}}^{2}-{B_{2}}^{2}+2\sqrt{3}A_{2}B_{2}+4{a_{2}}^{2}}}{2}\\ d_{3}=\frac{-\left(A_{3}-\sqrt{3}B_{3}\right)-\sqrt{-3{A_{3}}^{2}-{B_{3}}^{2}+2\sqrt{3}A_{3}B_{3}+4{a_{3}}^{2}}}{2} \end{array}$$

#### 2.2.2. Forward Kinematics Solution

The above equations represent the inverse kinematic analysis of the moving platform, resulting in the fundamental formula describing the relationship between the joints of the moving platform (Equation (8)). Thus, through input displacements (*d*_1_, *d*_2_, *d*_3_) along the three axes of the moving platform, the final position of the moving platform can be determined using the corresponding constraint relationship formulas. This constitutes the forward kinematics relationship. Equation (8) can hence be rewritten as:

(12) $$\begin{array}{l} {X_{D}}^{2}+{Y_{D}}^{2}+a_{11}X_{D}+a_{12}Y_{D}+a_{13}=0\\ {X_{D}}^{2}+{Y_{D}}^{2}+a_{21}X_{D}+a_{22}Y_{D}+a_{23}=0\\ {X_{D}}^{2}+{Y_{D}}^{2}+a_{31}X_{D}+a_{32}Y_{D}+a_{33}=0 \end{array}$$

where:

(13) $$\begin{array}{l} a_{11}=-2\left[\frac{\sqrt{3}}{3}\sin\left(60^{{}^{\circ}}-\phi \right)-\left(a_{1}-d_{1}\right)\right]\\ a_{12}=2\left[\frac{\sqrt{3}}{3}\cos\left(60^{{}^{\circ}}-\phi \right)-\frac{\sqrt{3}}{3}b_{1}\right]\\ a_{13}={\left[\frac{\sqrt{3}}{3}\sin\left(60^{{}^{\circ}}-\phi \right)-\left(a_{1}-d_{1}\right)\right]}^{2}+{\left[\frac{\sqrt{3}}{3}\cos\left(60^{{}^{\circ}}-\phi \right)-\frac{\sqrt{3}}{3}b_{1}\right]}^{2}-{a_{1}}^{2} \end{array}$$

Similarly, for the other two branched chains:

(14) $$\begin{array}{l} {X_{D}}^{2}+{Y_{D}}^{2}+a_{21}X_{D}+a_{22}Y_{D}+a_{23}=0\\ {X_{D}}^{2}+{Y_{D}}^{2}+a_{31}X_{D}+a_{32}Y_{D}+a_{33}=0 \end{array}$$

where:

(15) $$\begin{array}{l} \left(a_{11}-a_{21}\right)X_{D}+\left(a_{12}-a_{22}\right)Y_{D}+\left(a_{13}-a_{23}\right)=0\\ \left(a_{11}-a_{31}\right)X_{D}+\left(a_{12}-a_{32}\right)Y_{D}+\left(a_{13}-a_{33}\right)=0 \end{array}$$

(16) $$\begin{array}{l} a_{21}=2\left[\frac{\sqrt{3}}{3}b\sin\phi -b\cos\left(60^{{}^{\circ}}-\phi \right)-\left(\frac{a_{2}-b}{2}-\frac{d_{2}}{2}\right)\right]\\ a_{22}=2\left[-\frac{\sqrt{3}}{3}b\cos\phi +b\sin\left(60^{{}^{\circ}}-\phi \right)-\left(\frac{3\sqrt{3}a_{2}+\sqrt{3}b}{6}-\frac{\sqrt{3}}{2}d_{2}\right)\right]\\ a_{23}={\left[\frac{\sqrt{3}}{3}b\sin\phi -b\cos\left(60^{{}^{\circ}}-\phi \right)-\left(\frac{a_{2}-b}{2}-d_{2}\right)\right]}^{2}\\ +{\left[-\frac{\sqrt{3}}{3}b\cos\phi +b\sin\left(60^{{}^{\circ}}-\phi \right)-\left(\frac{3\sqrt{3}a_{2}+\sqrt{3}b}{6}-\frac{\sqrt{3}}{2}d_{2}\right)\right]}^{2}-{a_{2}}^{2}\\ a_{31}=2\left[\frac{\sqrt{3}}{3}b\sin\phi +b\cos\left(60^{{}^{\circ}}+\phi \right)-\left(\frac{a_{3}+b}{2}-\frac{d_{3}}{2}\right)\right]\\ a_{32}=2\left[-\frac{\sqrt{3}}{3}b\cos\phi +b\sin\left(60^{{}^{\circ}}+\phi \right)-\left(\frac{\sqrt{3}b-3\sqrt{3}a_{3}}{6}+\frac{\sqrt{3}}{2}d_{3}\right)\right]\\ a_{33}={\left[\frac{\sqrt{3}}{3}b\sin\phi +b\cos\left(60^{{}^{\circ}}+\phi \right)-\left(\frac{a_{3}+b}{2}-\frac{d_{3}}{2}\right)\right]}^{2}\\ +{\left[-\frac{\sqrt{3}}{3}b\cos\phi +b\sin\left(60^{{}^{\circ}}+\phi \right)-\left(\frac{\sqrt{3}b-3\sqrt{3}a_{3}}{6}+\frac{\sqrt{3}}{2}d_{3}\right)\right]}^{2}-{a_{3}}^{2} \end{array}$$

Equations (12)–(16) form a system of nonlinear equations, which, when solved, yield the following expressions for *X_D_* and *Y_D_*:

(17) $$\begin{array}{l} X_{D}=\frac{b_{1}}{b_{2}}\\ Y_{D}=\frac{b_{3}}{b_{4}} \end{array}$$

where

(18) $$\begin{array}{l} b_{1}=\left(a_{11}-a_{21}\right)\left(a_{12}-a_{32}\right)-\left(a_{11}-a_{31}\right)\left(a_{12}-a_{22}\right)\\ b_{2}=\left(a_{13}-a_{33}\right)\left(a_{12}-a_{22}\right)-\left(a_{13}-a_{23}\right)\left(a_{12}-a_{32}\right)\\ b_{3}=\left(a_{11}-a_{22}\right)\left(a_{11}-a_{31}\right)-\left(a_{12}-a_{32}\right)\left(a_{11}-a_{21}\right)\\ b_{4}=\left(a_{13}-a_{33}\right)\left(a_{11}-a_{21}\right)-\left(a_{13}-a_{23}\right)\left(a_{11}-a_{31}\right) \end{array}$$

Substituting Equation (18) into Equation (12) and collapsing it gives:

(19) $${\left(\frac{b_{2}}{b_{1}}\right)}^{2}+{\left(\frac{b_{4}}{b_{3}}\right)}^{2}+\frac{a_{11}b_{1}}{b_{2}}+\frac{a_{12}b_{3}}{b_{4}}+a_{13}=0$$

The *X_D_* and *Y_D_* values are thus obtained by substituting the obtained progeny into Equation (17).

## 3. Results and Discussion

### 3.1. Simulation

**Figure 6:**
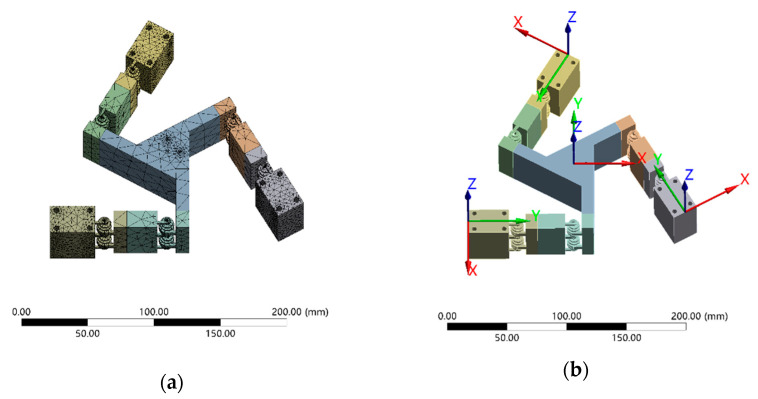
Finite element model of the planar 3-PRR flexible parallel mechanism’s micro-positioning stage: (**a**) finite element mesh and (**b**) displacement constraints.

A finite element model of the 3-PRR flexible parallel platform, which incorporates the mechanical structure shown in Figure 2 and the key dimensions listed in Table 1, was implemented with the commercial finite element analysis software ANSYS 19.2. It is worth noting that the specified dimensions may be adjusted to suit alternative applications. The hinge was additively manufactured using a photopolymer with Young’s modulus of 2.2 GPa, density of 1.12 kg/m^3^, and Poisson’s ratio of 0.42. The entire moving platform was discretized using Solid95 elements. To enhance the accuracy of the finite element solution, a refined mesh was assigned to the thin and non-stationary components. The resultant finite element model is shown in Figure 6a.

**Figure 7:**
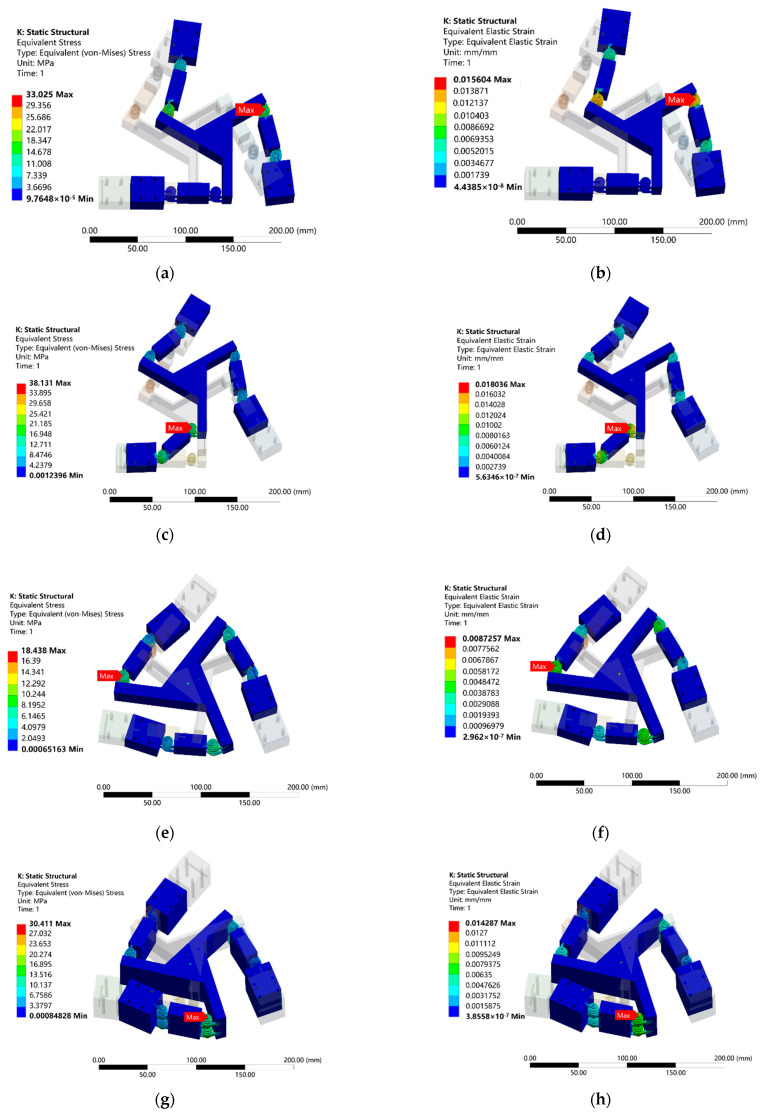
Contour maps obtained from the simulation of the proposed platform: (**a**) stress and (**b**) strain for a translation of 35 mm along the X-axis; (**c**) stress and (**d**) strain for a translation of 35 mm along the Y-axis; (**e**) stress and (**f**) strain for a rotation of 35° around the Z-axis; (**g**) stress and (**h**) strain for a motion of 25.5 mm, −15.43 mm, 26.2°.

An inverse kinematic analysis was conducted based on simulations, with the centroid of the moving platform serving as the primary driver. The maximum displacement or rotation of the moving platform along each axis was obtained by appropriately constraining the platform and varying the target motion parameter (movement along the X- or Y-axis or rotation around the Z-axis) while ensuring that the hinge remained below its yield stress. In each motion chain of the platform, restrictions were applied to the three axes, allowing movement only along the guiding direction, as shown in Figure 6b. The resultant changes in the platform displacement and orientation were obtained by altering the input displacement values for the three axes. Table 2 presents a comparison between the simulated and theoretical kinematic results. As shown in Figure 7, the maximum stress observed when moving the platform to its maximum displacement along any of the tested axes was 38 MPa, which was significantly less than 67 MPa, the yield stress of the material.

The first step for performing forward kinematic analysis involved establishing an appropriate coordinate system and constraining the motion of each of the three links, *A*_1_*B*_1_, *A*_2_*B*_2_, and *A*_3_*B*_3_, to ensure that the platforms moved only along their respective guiding directions, as depicted in Figure 6b. Next, the resultant changes in displacement and angle of the dynamic platform were determined by adjusting the input displacement values of the three links. Subsequently, these input displacement values were substituted into the forward kinematic modeling equations. Finally, the equation results were compared with those of the finite element simulation, as presented in Table 2.

The first step to performing inverse kinematic analysis involved defining an appropriate coordinate system and constraining the parallel motion platform to move only along the guiding directions of the three links *A*_1_*B*_1_, *A*_2_*B*_2_, and *A*_3_*B*_3_. Subsequently, the resultant displacements along the three axes were obtained by using the center of mass of the moving platform as the driving factor and setting different motion parameters (movement along the X- and Y-axes and rotation around the Z-axis). Next, these motion parameters were substituted into the inverse kinematic modeling equations. Finally, the equation results were compared with those of the finite element simulation, as presented in Table 3.

The comparison between the results of the finite element analysis and those of the theoretical model (Table 2 and Table 3) revealed a maximum error of 5.4%, thus validating the accuracy of the theoretical model. This error could be potentially attributed to parasitic motion, with the simulation considering additional compression in the flexible hinges. Furthermore, in the finite element model, the entire platform is defined as a flexible body, considering shear in the calculation process; in contrast, in the theoretical model, the flexible hinge is assumed to be elastic, whereas the remaining parts are treated as rigid components, leading to a discrepancy in the primary data. In addition, the meshing and node selection during the finite element model simulation may have affected the calculation results.

### 3.2. Experiments

**Figure 8:**
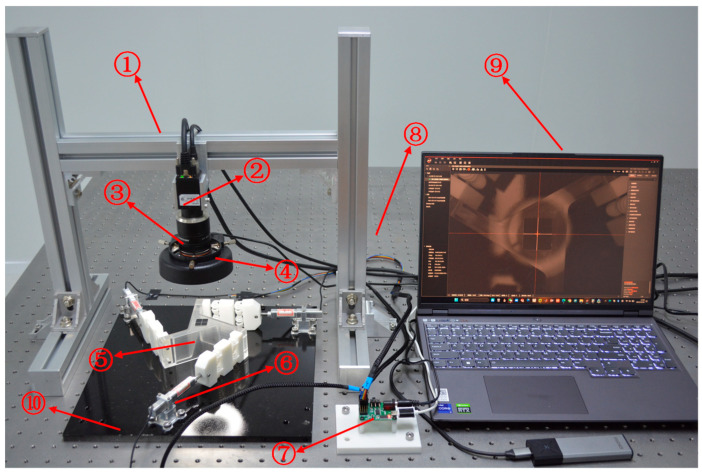
Experimental setup: 1—gantry, 2—industrial camera, 3—high-resolution lens, 4—cold light source, 5—3-PRR parallel platform, 6—micro linear servo drive, 7—signal acquisition module, 8—air-floating isolation platform, 9—PC, and 10—base plate.

**Figure 9:**
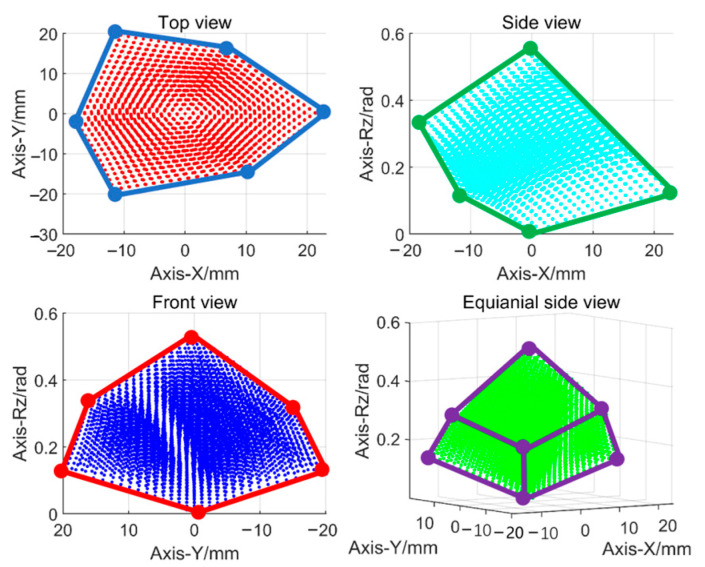
Workspace of the planar 3-PRR flexible parallel mechanism.

A prototype of the proposed large-stroke 3-PRR parallel platform, based on biomimetic variable-diameter spiral flexible hinges, was fabricated to assess its practical performance. Hinges were additively manufactured using a photosensitive polymer. An experimental setup was constructed, as shown in Figure 8, comprising a gantry, three-degree-of-freedom positioning platform, air-floating isolation platform, industrial camera, light sources, micro linear servo drives, a signal acquisition module, and a PC. The three micro linear servo drives (CM-LA30-021D) were controlled in real time using the PC. The parallel platform was mounted on a base plate that was securely fixed to an air-floating isolation platform to mitigate external disturbances. The industrial camera (HIKROBOT, MV-CS200-10GM, pixel size: 2.4 μm × 2.4 μm, resolution: 5472 × 3648, lens: HIKROBOT MVL-KF1624M-25MP, focal length: 16 mm) served as an external sensor and was positioned on the gantry directly above the moving platform. The displacement of the platform was determined by processing each pair of consecutive photographic frames captured by the industrial camera. As depicted in Figure 9, the workspace of the motion platform was determined using the inverse kinematics solution formula and processed with the commercial MATLAB R2021a software. Selecting any boundary point within the workspace as the input for the motion platform allowed for the determination of the actual workspace. This mechanism achieved pure motions of 30 mm along the X- and Y-axes as well as pure rotation of 35° around the Z-axis.

**Figure 10:**
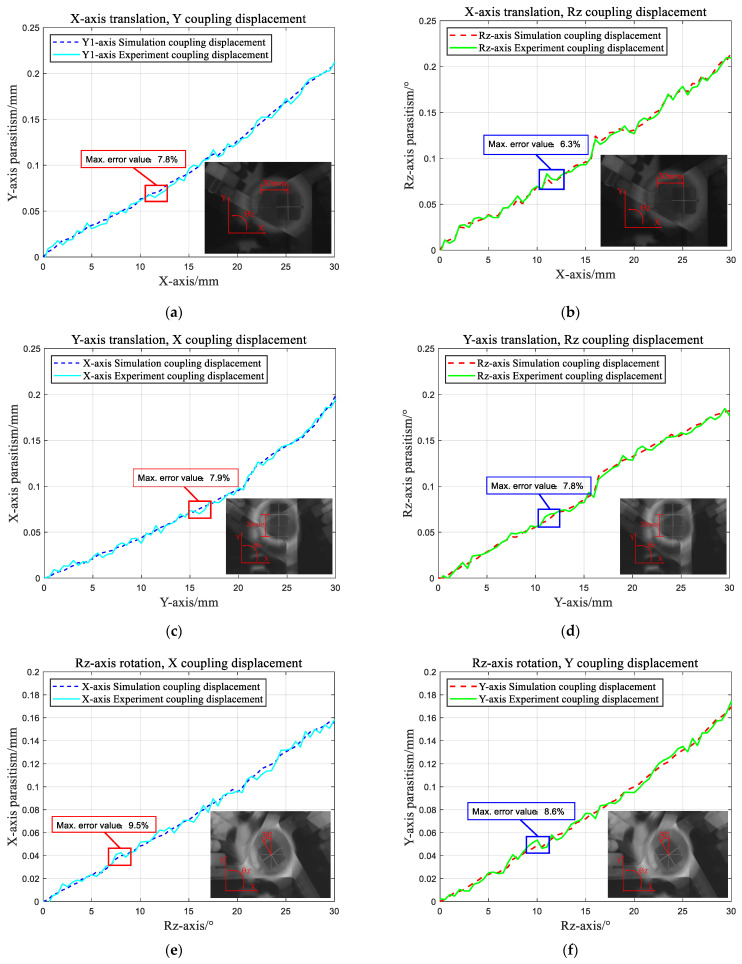
Parasitic motion of the moving platform prototype: (**a**) Y-axis and (**b**) Z-axis coupled displacements during pure X-axis translation; (**c**) X-axis and (**d**) Z-axis coupled displacements during pure Y-axis translation; and (**e**) X-axis and (**f**) Y-axis coupled displacements during pure Z-axis rotation.

An experiment was conducted to investigate parasitic motion while attempting to achieve pure motion along each axis. Open-loop control of the micro linear servo drives was utilized to attain pure horizontal movement of the motion platform along the X- and Y-directions and pure rotation around the Z-axis by controlling the inputs of the three chains. However, during each pure motion of the platform, coupled motions were observed in the remaining directions. Using the experimental setup depicted in Figure 8, images of the motion platform before and after motion were processed to determine the maximum coupled displacement in the static directions. As shown in Figure 10, during pure motion in the X-direction, the maximum coupled errors in the Y- and Z-directions were 7.8% and 6.3%, respectively. Similarly, during pure motion in the Y-direction, the maximum coupled errors in the X- and Z-directions were 7.9% and 7.8%, respectively. Finally, when undergoing pure motion in the Z-direction, the maximum coupled errors in the X- and Z-directions were 9.5% and 8.3%, respectively. The linear modeling of flexible elements in the theoretical model and the idealization of the flexible mechanism as rigid/flexible can be regarded as the main contributors to the errors observed between the experimental and theoretical calculations. Moreover, parasitic hinge motions may occur due to the additional compression exerted in other directions on the flexible hinges during the experimental process. Additionally, errors incurred during the printing process, which alter the mechanisms’ dimensions, further contribute to the overall experimental errors. The experimental data indicate that the maximum error is 9.5%, falling within an acceptable range. Thus, the experimental results can be considered to closely align with the theoretical model, thereby validating the accuracy of the latter. For each axis of directed motion, Figure 8 shows the resulting maximum parasitic motion along the other two axes. The mechanism achieved a maximum pure motion of 30 mm along the X- and Y-axes and a pure rotational motion of 35° around the Z-axis.

The compactness of the proposed micro-positioning platform was compared with that of previously designed systems, as shown in Table 4. A compactness index based on the workspace size was defined as follows:

$$\mathrm{Compactness}\;\mathrm{Index}=\frac{\mathrm{Workspace}\;\mathrm{Volume}}{\mathrm{Mechanism}\;\mathrm{Volume}}\times 10^{4}$$

The results demonstrated that the proposed mechanism has a higher compactness index than other mechanisms proposed in the literature.

## 4. Conclusions

The large size, slow motion, and low precision of traditional three-degree-of-freedom XYƟz motion platforms limit their application in critical scenarios, particularly where ultra-high-precision is required over wide ranges of motion and within a small footprint. Hence, in this study, we proposed an integrated planar three-degree-of-freedom compliant parallel positioning platform using custom biomimetic flexible hinges. The platform is driven by miniature linear servo actuators and utilizes a flexible hinge transmission, where each hinge is capable of large rotational movements, enabling far-reaching multi-degree-of-freedom positioning within a limited spatial envelope. A rigid-body model was developed for the platform, and a kinematic analysis was performed to obtain both forward and inverse kinematic theoretical models. Thes models were validated against finite element simulation results, yielding a maximum error of 5%. Subsequently, a prototype of the system was fabricated, and its performance was compared with that obtained from the finite element analysis, revealing maximum errors of 7.8%, 7.9%, and 9.5% for the X- and Y-displacements and Z-rotational angle, respectively. This validates the feasibility of the kinematic modeling approach. Furthermore, in comparison with other systems documented in the literature, the proposed platform demonstrated a superior “compactness index,” defined as the ratio of workspace to mechanism volumes. Our future endeavors will focus on establishing a closed-loop system for the motion platform with the objective of further enhancing its mechanical performance and minimizing the coupled errors in static directions.

## Figures and Tables

**Table 1:** Key dimensions of the flexible mechanism.

Parameter	*L*	*W*	*H*	*w*	*t*	*an*	$a_{i}(i=1,2,3)$	*b*
Value (mm)	44	14	14	2	2	7	45	100

**Table 2:** Comparison of theoretical and simulated kinematic forward solution analysis results.

Test Number	Input Displacement (mm)			Simulation Output Displacement (mm-deg)			Theoretical Output Displacement (mm-deg)			Error (%)		
*i*	*d*_1_	*d*_2_	*d*_3_	*X_P_*	*Y_P_*	*θ_P_*	*X_P_*	*Y_P_*	*θ_P_*	*e_X_* *_p_*	*e_Y_* *_p_*	*e_d_*_3_
1	−20	3	6	−14.74	1.95	−4.68	−15.3	1.94	−4.95	3.5	0.5	5.4
2	10	−5	10	11.93	−2.66	−2.59	12.42	−2.54	−2.69	3.9	4.7	3.7
3	10	10	10	0.185	0.185	9.91	0.173	0.174	10.15	6.9	6.3	1.8
4	−18	13	−25	−8.73	−22.89	−12.9	−8.25	−23.3	−12.85	5.8	1.6	0.39
5	−5	−10	−5	1.69	2.88	−6.58	1.62	2.86	−6.67	4.3	0.69	1.3

**Table 3:** Comparison of theoretical and simulated kinematic inverse solution analysis results.

Test Number	Displacement of the Platform (mm)			Simulation Input Displacement (mm-deg)			Theoretical Input Displacement (mm-deg)			Error (%)		
*i*	*X_P_*	*Y_P_*	*θ_P_*	*d*_1_	*d*_2_	*d*_3_	*d*_1_	*d*_2_	*d*_3_	*e_d_*_1_	*e_d_*_2_	*e_d_*_3_
1	28	21	5.7	38.1	−24.45	22.02	39.11	−24.17	23.21	2.5	1.1	5.1
2	−13	15	1.17	−9.02	−1.25	21.54	−9.26	−1.24	20.8	2.5	0.8	3.1
3	−28	15	−11	−36.7	1.74	18.78	−36.5	1.67	19.33	0.57	4.1	2.8
4	15	5	10	26.3	−0.39	9.5	25.38	−0.377	9.266	3.6	3.4	2.5
5	−5	−5	−5	0.31	10.5	3.5	0.296	10.91	3.62	4.7	3.8	3.3

**Table 4:** Compactness of various micro-positioning systems.

Design	Mechanism Volume (mm^3^)	Workspace Volume (mm^2^-mrad)	Compactness Index (rad/mm)
[10]	156.48 × 137.51 × 92.5	2.86 × 3.22 × 66.29	3.0672
[10]	350 × 350 × 60	1.2 × 1.2 × 104.72	0.2052
[21]	350 × 270 × 60	2.5 × 2.5 × 174.53	1.8967
[22]	400 × 400 × 55	5 × 5 × 87.27	2.4716
This work	287 × 275 × 46	30 × 30 × 610.86	1514

## Data Availability

The data presented in this study are available upon request from the corresponding author. Specific experimental data have been published in the article.
